# Effectiveness of Intravenous Immunoglobulin for Management of Pain in Patients with Postpolio Syndrome

**DOI:** 10.1155/2021/6637705

**Published:** 2021-03-20

**Authors:** Min Cheol Chang, Jin-Sung Park, Jong-moon Hwang, Donghwi Park

**Affiliations:** ^1^Department of Rehabilitation Medicine, College of Medicine, Yeungnam University, Daegu, Republic of Korea; ^2^Department of Neurology, School of Medicine, Kyungpook National University, Kyungpook National University Chilgok Hospital, Daegu, Republic of Korea; ^3^Department of Rehabilitation Medicine, School of Medicine, Kyungpook National University, Daegu, Republic of Korea; ^4^Department of Physical Medicine and Rehabilitation, Ulsan University Hospital, University of Ulsan College of Medicine, Ulsan, Republic of Korea

## Abstract

**Objective:**

Many patients with postpolio syndrome (PPS) experience pain. In this study, we aimed to review previous studies to investigate the effectiveness of intravenous immunoglobulin (IVIG) for managing pain in patients with PPS. We performed a narrative review.

**Methods:**

In PubMed, we searched for the keywords ((Immunoglobulin OR IVIG) AND (poliomyelitis OR poliomyelitis syndrome)). We included articles in which IVIG was infused in patients with PPS and pain severity was measured before and after treatment.

**Results:**

In the results, five articles (4 randomized controlled trials and 1 prospective observational study) were included in this review. Four of the studies reported that IVIG had a positive pain-reducing effect in patients with PPS. In addition, 4 studies evaluated the outcomes related to muscle strength and function. Of these studies, 3 showed some improvement in measurements for muscle strength and function.

**Conclusion:**

In conclusion, IVIG might be one of the beneficial options for managing pain in PPS. Pain reduction might be responsible for the improvement of muscle strength or function. To confirm the benefits of IVIG in reducing pain, more high-quality studies are required.

## 1. Introduction

Postpolio syndrome (PPS) was reported in survivors from acute poliomyelitis infection [1]. The prevalence of polio survivors with PPS ranged from 20% to 80%, depending on the study population and diagnostic criteria [2]. PPS appears several decades after a period of stability. The symptoms of PPS include muscle weakness, fatigue, and pain [3, 4]. Ongoing denervation is known to be the most important cause of muscle weakness from PPS [3, 4]. Denervation is compensated for by reinnervation by collateral sprouting, but in the end, reinnervation cannot keep up with speed of denervation, which leads to insufficient compensation and causes muscle weakness [3, 4].

Pain is one of the common complaints of patients with PPS, which decreases quality of life and hinders activities of daily living [5–9]. The most commonly occurring pain is muscle or joint pain in the lower extremity or lower back [7]. Muscle pain is caused by overuse of weak muscles or other muscles that compensate for the weakened polio-affected muscles [7]. Joint pain is caused by abnormal biomechanics, leg-length discrepancy, and muscle atrophy [7]. Neuropathic pain can also develop from neuroinflammation induced by poliomyelitis infection and a concomitant disorder in the nervous system, such as nerve impingement and herniated lumbar disk [7, 10].

The degree of pain from PPS is moderate to severe [7]. For managing pain in patients with PPS, oral drugs, physical therapies, and treatment procedures can be applied [11–13]. However, despite these treatments, pain can be unendurable. In addition, the effects of these treatments are sustained only for a short time. Some studies reported the positive pain-reducing effect of intravenous immunoglobulin (IVIG) [14–16]. IVIG might be one of the useful options for alleviating pain caused by PPS.

Here, we reviewed previous studies to investigate the effectiveness of IVIG for managing pain in patients with PPS.

## 2. Methods

Two authors (D. P. and M. C. C.) independently performed a literature search in PubMed. Differences in their search results were resolved through a discussion. The keywords used in the search were ((Immunoglobulin OR IVIG) AND (poliomyelitis OR poliomyelitis syndrome)). The search was limited to articles published up to June 27, 2020. The following inclusion criteria were applied for the selection of articles: (1) IVIG was infused in patients with PPS, (2) pain severity was measured before and after treatment, and (3) written in English. We excluded case reports and reviews. Data were extracted by two independent reviewers (D. P. and M. C. C.).

## 3. Results

A total of 1,941 potentially relevant articles were found in the primary literature search. After reading the titles and abstracts and assessing them for eligibility based on the full-text articles, 5 articles were finally included in this review (Table 1). Of the 5 previous studies [10, 17–20], 4 were randomized controlled trials (RCTs) [17–20], and one was a prospective observational study [10].

The first study on the pain-reducing effect of IVIG in patients with PPS was performed by Gonzalez et al. in 2006 [17]. They recruited 142 patients with PPS from 4 university clinics and randomly allocated 73 patients in the IVIG group, who received a total of 90 g of IVIG for 3 consecutive days and repeated 3 months later, and 69 patients in the placebo group, who received a total of 1800 mL of placebo saline for 3 consecutive days and repeated 3 months later. Three months after the second infusion, the decrement in the visual analogue scale (VAS) score (range: 0–10, where 0 indicates no pain and 10 indicates the worst imaginable pain) was not significantly different between the two groups. However, the median muscle strength was greater in the IVIG group by 8.6% than that in the placebo group. The quality of life after the treatment that was measured with the Short Form-36 (SF-36) questionnaire was not significantly different between the 2 groups.

In contrast to the study of Gonzalez et al. [17], the other 4 studies [10, 18–20] showed a positive pain-reduction effect after IVIG infusion in patients with PPS. In 2007, Farbu et al. [18] recruited 20 patients with PPS and equally allocated them into two groups (IVIG group: 10 patients; placebo group: 10 patients). IVIG was administered at a dose of 2 g/kg of body weight for 2–4 days. The placebo group received normal saline infusion at a volume corresponding to that of IVIG. They reported that IVIG effectively alleviated pain relative to placebo 3 months after the treatment (IVIG group vs. placebo group: VAS score, from 4.5 to 2.9 vs. from 4.6 to 6.1). However, the pain-reduction effect disappeared during the next 3 months. In contrast to the pain-reduction effect, no improvements in fatigue and muscle strength were observed.

In 2012, Gonzalez et al. [19] recruited 41 patients, allocating 20 patients to the IVIG group and 21 patients to the placebo group. To the patients in the IVIG group, 90 g of IVIG was given and repeated 3 months later. In the patients in the placebo group, a placebo solution containing glucose and water was infused. Gonzalez et al. reported that the VAS score significantly decreased 1 year after the treatment in the IVIG group as compared with the placebo group (mean VAS score: from 3.1 to 2.3 vs. from 3.1 to 2.9). Walking ability and quality of life were also significantly improved in the IVIG group. Furthermore, they found that the IFN-*γ* and IL-23 levels in cerebrospinal fluid were decreased in the IVIG group.

In 2013, Bertolasi et al. [20] found that pain intensity measured using the VAS decreased by approximately 1 point each in 24 patients who received IVIG therapy (0.4 g/kg/day for 5 consecutive days) at the 2- and 4-month follow-ups after therapy completion, but 26 patients in the placebo group (3000 mL placebo saline for 5 consecutive days) showed decreases in VAS scores of only 0.5 and 0.1 at the 2- and 4-month follow-ups, respectively. In their study, although the decrease in the VAS score was greater in the IVIG group, a statistical analysis was not performed. In addition, at the 2-month follow-up, the outcome according to the SF-36 quality-of-life measurement was significantly improved in terms of the items “role physical” and “role emotional.”

In another prospective observational study, Werhagen and Borg [10] demonstrated the significant pain-reducing effect of IVIG. They recruited 45 patients with PPS who had pain, including neuropathic pain (1 patient), nociceptive pain (39 patients), and mixed pain (5 patients). All the patients received an infusion of a total of 90 g of IVIG over 3 consecutive days. At the 6-month follow-up after the treatment, 31 patients (69%) showed pain reduction from a mean VAS score of 5.3 to 4.2. Furthermore, 18 patients (40%) had a decrease in the VAS score of ≥2 points.

## 4. Discussion

Of the 5 previous studies [10, 18–20], except 1 study [17], IVIG significantly alleviated pain in patients with PPS. We think that the results of these studies suggest that IVIG might be a good therapeutic option for alleviating pain due to PPS. Furthermore, 4 studies [17–20], excluding the study of Werhagen and Borg [10], evaluated the effect of IVIG on muscle strength or motor function. Of these 4 studies [17–20], excluding the study of Farbu et al. [18], 3 showed improvement of outcomes related to motor strength or function.

The mechanism of pain reduction of IVIG in patients with PPS has not been clearly elucidated. However, we propose some possible mechanisms. The levels of several cytokines are increased in the cerebrospinal fluid of patients with PPS, which activates microglia and macrophages, generating noxious effects on neural cells by producing nitric oxide, protease, and glutamate products [19, 21]. IVIG infusion is reported to reduce the levels of some cytokines [19]. This effect of IVIG seems to protect the neural cells, which appear to contribute to the improvement of motor weakness or function. The enhanced muscle strength or motor function would prevent overuse of specific muscles and, thus, might reduce the occurrence of pain in overused muscles and joints. In addition, elevated cytokine levels in cerebrospinal fluid seem to be associated with the occurrence of neuropathic pain in patients with neurological disorders [22, 23]. The reduction of cytokine levels after IVIG infusion might play a role in alleviating neuropathic pain [19].

The 5 previous studies that evaluated the pain-reducing effect of IVIG did not investigate nociceptive pain and neuropathic pain separately [10, 17–20]. Therefore, we cannot identify which types of pain would have a good response to the pain-reducing effect of IVIG infusion. In the future, RCTs that assess nociceptive and neuropathic pain separately should be conducted.

In conclusion, this review shows that IVIG administration might be useful for managing pain in patients with PPS. In the 5 articles reviewed, no major adverse effects were reported. Owing to the small number of studies and inconsistent results between the studies, we cannot make a definite conclusion on the effect of IVIG on pain in PPS. However, we think that the previous studies showed a possibility that IVIG can safely manage pain in patients with PPS. To confirm the benefits of IVIG in reducing pain in patients with PPS, more high-quality studies are required. Moreover, the protocols for IVIG infusion used in each study were heterogeneous; therefore, the most effective protocol for IVIG infusion for controlling pain should be evaluated in the future.

## Figures and Tables

**Table 1:** Summary of the included studies.

#	First author, year	Study design	Number of patients (E/C)	Treatment compared with IVIG	IVIG protocol	Summary of the outcome
1	Gonzalez, 2006 [17]	RCT	142 (73/69)	Placebo	30 g^*∗*^3 days (repeated after 3 months)	No significant effect

2	Farbu, 2007 [18]	RCT	20 (10/10)	Placebo	2 g/kg for 2–4 days	At the 3-month follow-up, IVIG group > placebo group, IVIG group: VAS 4.5 (pretreatment) ⟶ 2.9 (3 months)

3	Werhagen, 2011 [10]	Single-arm prospective study	45	—	30 g^*∗*^3 days	After IVIG, VAS 5.3 (pretreatment) ⟶ 4.2 (6 months)

4	Gonzalez, 2012 [19]	RCT	41 (20/21)	Placebo	90 g (repeated after 3 months)	IVIG group > placebo group; after IVIG, VAS 3.1 (pretreatment) ⟶ 2.3 (1 year)

5	Bertolasi, 2013 [20]	RCT	50 (24/26)	Placebo	0.4 g/kg/day for 5 days	IVIG: VAS 5.4 (pretreatment) ⟶ 4.4 (2 months); placebo: VAS 4.9 (pretreatment) ⟶ 4.4 (2 months)

E: experimental group; C: comparison group; IVIG: intravenous immunoglobulin; RCT: randomized controlled trial; VAS: visual analogue scale.
